# Novel technique for management of Bouveret syndrome

**DOI:** 10.1016/j.vgie.2023.06.006

**Published:** 2023-08-04

**Authors:** Angelo S.T. Kum, Sultan Mahmood, Erik Holzwanger, Samuel Igbinedion, Moamen Gabr, Mandeep S. Sawhney, Tyler M. Berzin, Douglas K. Pleskow

**Affiliations:** Center for Advanced Endoscopy, Division of Gastroenterology, Beth Israel Deaconess Medical Center, Harvard Medical School, Boston, Massachusetts

## Abstract

Video 1 Endoscopic treatment of a Bouveret syndrome showing and describing the techniques and procedures involved.

Endoscopic treatment of a Bouveret syndrome showing and describing the techniques and procedures involved.

## Case Description

**Figure 1:**
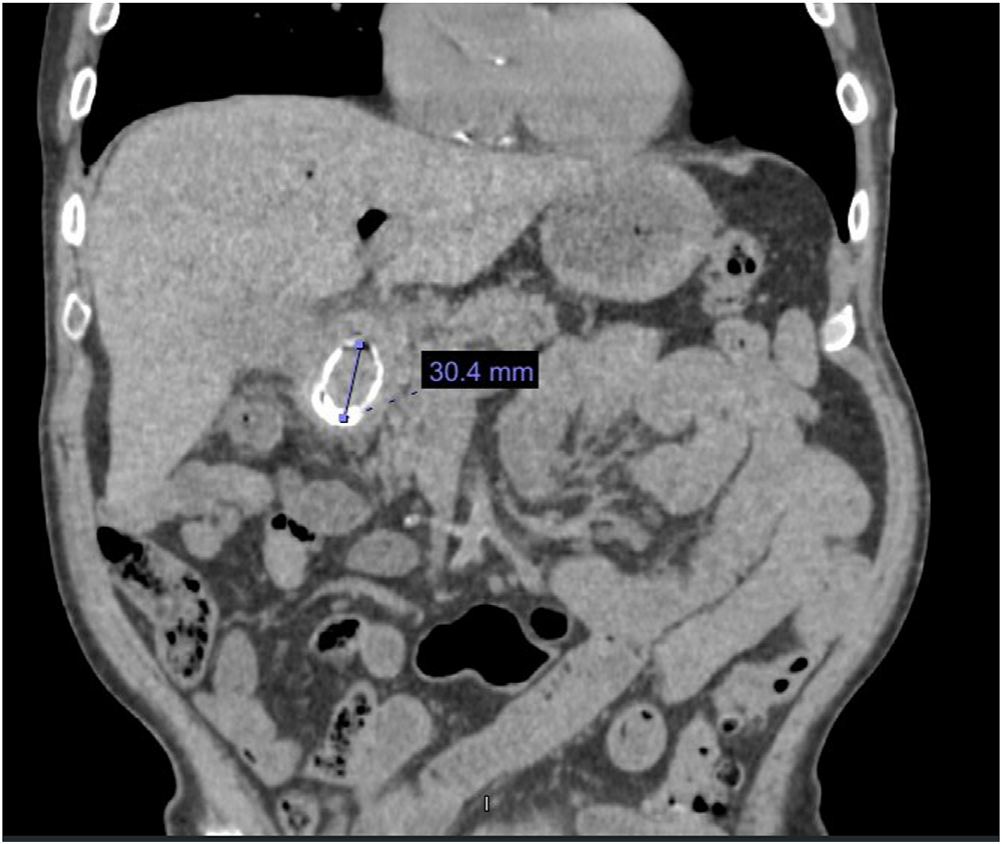
Abdomen CT scan result showing a 30.4-mm stone in the descending duodenum and intrahepatic biliary air, indicating a bilioenteric fistula.

A 76-year-old man with a history of coronary artery disease with percutaneous coronary intervention, pacemaker, and diabetes presented to the emergency department with sudden onset of abdominal pain and vomiting. The CT abdomen scan (Fig. 1) showed a 30.4-mm stone in the descending duodenum and intrahepatic biliary air, indicating a bilioenteric fistula. The diagnosis of Bouveret syndrome (BS) was made. BS is a rare cholelithiasis adverse event with bilioenteric fistula and gallstone migration, promoting gastric outlet obstruction symptoms. An endoscopic approach can be a definitive therapy for most patients.^1^ Although mechanical or electrohydraulic lithotripsy may reduce the size of the gallstone, fragmented pieces may struggle to pass through the cricopharyngeal sphincter. A novel technique with a modified overtube was used to remove the stone safely.

## Endoscopic Procedure

An EGD with a clear distal attachment was used to identify the location and aspect of the gallstone (Video 1, available online at www.videogie.org). We used a snare to fragment the gallstone impacted in the duodenal bulb, reducing the original size of 30.4 mm to approximately 20 mm, followed by retrieval into the stomach with a Roth net (STERIS Endoscopy, Mentor, Ohio, USA). Electrohydraulic lithotripsy (EHL) was performed to reduce the stone size. However, this was unsuccessful because of the unstable scope positioning and stone mobility, which could have been solved if the EHL was performed in the bulb. We attempted to remove the stone with a Roth net without success as the stone would impact at the level of the upper esophageal sphincter. Another attempt of mechanical lithotripsy with a basket resulted in a broken handle cord because of the gallstone hardness. This corroborates the radio-opaque CT findings suggesting a pigment stone composed of calcium bilirubinate. We then used a modified technique by capturing the stone with a snare and an adapted overtube, which had its distal tip cut longitudinally to create a flap and adjust the stone into the tube.^2^ This adaptation provided a better fit for the stones larger than the 16.7 mm of the regular overtube diameter, leading to a more secure and safe removal through the upper esophageal sphincter. Complete removal of the stone was achieved with gentle manipulation. At the end of the procedure, the duodenum was re-evaluated and no obvious deformity was observed.

## Outcome

The patient had complete resolution of the GI symptoms after the endoscopic procedure, and a cholecystectomy with surgical repair of the cholecystoduodenal fistula was performed.

## Conclusions

Endoscopy for BS is a well-tolerated treatment option. EHL may be performed with more stability in the duodenal bulb, but it can be difficult to perform in the stomach. Pigment gallstones seen on CT can be very hard and resistant to mechanical lithotripsy. The upper esophageal sphincter can become a barrier for successful removal of the gallstone, and the overtube adaptation with a flap on the tip helps to fit larger stones and to remove them safely through the upper esophageal sphincter.

## Disclosure

*Dr Holzwanger is a consultant for Virgo. Dr Gabr is a consultant for Medtronic, ConMed, Olympus, Boston Scientific, Fuji, and Adaptive Endo. Dr Berzin is a consultant for Boston Scientific, Medtronic, and Fuji. Dr Pleskow is a consultant for Boston Scientific and Olympus. The other authors did not disclose any financial relationships.*

## Supplementary data

Video 1 Endoscopic treatment of a Bouveret syndrome showing and describing the techniques and procedures involved.
